# Deep and surface learning in problem-based learning: a review of the literature

**DOI:** 10.1007/s10459-015-9645-6

**Published:** 2015-11-13

**Authors:** Diana H. J. M. Dolmans, Sofie M. M. Loyens, Hélène Marcq, David Gijbels

**Affiliations:** 1 Department of Educational Development and Research, School of Health Professions Education (SHE), Faculty of Health, Medicine and Life Sciences (FHML), Maastricht University, PO Box 616, 6200 MD Maastricht, Netherlands; 2 Institute of Psychology, Erasmus University Rotterdam, Rotterdam, Netherlands; 4 Management of Learning, Maastricht University, Maastricht, Netherlands; 5 Department of Training and Education, EduBROn research group, Faculty of Social Sciences, University of Antwerp, Antwerp, Belgium; 6 Roosevelt Center for Excellence in Education, University College Roosevelt, Middelburg, Netherlands

**Keywords:** Problem-based learning, Deep approach, Surface approach, Students’ approaches to learning (SAL)

## Abstract

In problem-based learning (PBL), implemented worldwide, students learn by discussing professionally relevant problems enhancing application and integration of knowledge, which is assumed to encourage students towards a deep learning approach in which students are intrinsically interested and try to understand what is being studied. This review investigates: (1) the effects of PBL on students’ deep and surface approaches to learning, (2) whether and why these effects do differ across (a) the context of the learning environment (single vs. curriculum wide implementation), and (b) study quality. Studies were searched dealing with PBL and students’ approaches to learning. Twenty-one studies were included. The results indicate that PBL does enhance deep learning with a small positive average effect size of .11 and a positive effect in eleven of the 21 studies. Four studies show a decrease in deep learning and six studies show no effect. PBL does not seem to have an effect on surface learning as indicated by a very small average effect size (.08) and eleven studies showing no increase in the surface approach. Six studies demonstrate a decrease and four an increase in surface learning. It is concluded that PBL does seem to enhance deep learning and has little effect on surface learning, although more longitudinal research using high quality measurement instruments is needed to support this conclusion with stronger evidence. Differences cannot be explained by the study quality but a curriculum wide implementation of PBL has a more positive impact on the deep approach (effect size .18) compared to an implementation within a single course (effect size of −.05). PBL is assumed to enhance active learning and students’ intrinsic motivation, which enhances deep learning. A high perceived workload and assessment that is perceived as not rewarding deep learning are assumed to enhance surface learning.

## Introduction

Universities are facing challenges today in educating students to become life-long learners and versatile experts in their own fields. Fostering and stimulating the development of lifelong learning skills such as problem solving and critical thinking has become a crucial goal of higher education in the twentyfirst century. According to the Bologna declaration, successful learning and studying in higher education should involve students in deep learning (Asikainen 2014). In a recent literature review, Dinsmore and Alexander (2012) state that if research on students’ learning is going to have any bearing on practice, one area in need of critical discussion is the investigation of deep and surface learning. From their review, Dinsmore and Alexander (2012) identified why the results of studies on deep and surface learning often result in ambiguous and inconsistent findings. One of the reasons is that the conceptualization of deep and surface learning differs across studies as well as the way in which these concepts are measured. Often evidence of the validity of the instruments used to measure deep learning is lacking. Another reason is that the contexts in which the studies are conducted often vary, whereas deep learning might differ across contexts and academic domains. As a consequence, Dinsmore and Alexander (2012) emphasized from their review that it is important in future research to (a) clearly define what is meant by deep learning, starting from a clear theoretical framework, (b) investigate deep learning within a specific learning context, since the context of the learning environment may influence deep learning, and (c) measure deep learning by means of valid tools. In the present review-study, we aim to take these recommendations into account.

Below we will first explain the framework of students’ approaches to learning (SAL) as the theoretical framework guiding this study. Next, we will elaborate on problem-based learning (PBL) as the learning context under study. Finally, we will explain how we have taken the issue of valid tools into account and we will present the central research questions of this review.

### Students’ approaches to learning as a theoretical framework

Theoretically, we build on the framework of students’ approaches to learning (SAL). The concept of a deep approach to learning originated in the work of Marton and Säljö (1976). They discovered that students had different intentions when approaching a particular task (i.e., studying a text for later use). Some students intended to understand the meaning of the text, while others primarily wanted to be able to reproduce what they had read when questioned on it. Students with an intention to extract meaning from their readings were likely to try to relate information to prior knowledge, to structure ideas into comprehensible wholes, and to critically evaluate knowledge and conclusions presented in the text. Students who took upon themselves the task of committing text to memory were likely to use processing strategies such as rote learning. The former combination of intentions and processing strategies became known as a deep approach to learning and the latter as a surface approach. Trigwell et al. (2005) argue that students with a deep approach to learning are intrinsically interested and try to understand what they study. Students adopting a surface approach mainly focus on rote learning and primarily study to pass the test. Deep and surface approaches to learning are seen as a combination of students’ intentions (or motives) and the accompanying learning activities. A surface approach to learning has typically been defined as an intention to reproduce content, with learning processes characterized by rote learning and memorization. A deep approach to learning has been described as a student’s intention to understand content together with the processes of relating and structuring ideas, looking for underlying principles, weighing relevant evidence, and critically evaluating knowledge (Biggs et al. 2001; Entwistle and McCune 2004; Lonka and Lindblom-Ylänne 1996; Loyens et al. 2013). Approaches to learning are assumed to be related to the perceived demands of the learning environment and are not seen as purely personal characteristics (Biggs and Tang 2007; Nijhuis et al. 2005). How students approach their learning is viewed as changeable and influenced by factors in the learning environment, students’ perceptions of these factors and student characteristics such as their prior knowledge on the topic under study (Gijbels et al. 2014). This is where the concept of approaches to learning differs from the concept of learning styles in which all learners are claimed to have their own personal and stable learning style that should be aligned to instruction. The field of learning styles has recently been heavily critiqued because of the lack of solid evidence that learning styles—as stable individual characteristics—actually exists (see e.g. Kirschner and Van Merriënboer 2013). However, research that investigated the kind of learning approaches that are used by students in university education has led to contradictory results (see e.g., Gijbels et al. 2009; Struyven et al. 2006; Wilson and Fowler 2005). Baeten et al. (2010) reviewed 25 studies to detect which factors encourage or discourage a deep approach to learning in student-centered learning environments in general. Their review demonstrated that characteristics of the teaching method, how students perceive the teaching context, and student factors play a role. Baeten et al. (2010) concluded that many of these factors are intertwined and that still little is known about how they relate to each other and differ across different student-centered learning environments. The aim of the present paper is to overcome this problem of inconsistency and ambiguity in the empirical research on deep and surface approaches to learning and contribute to our understanding of students’ learning in higher education. Numerous attempts have been made to optimize students’ learning in higher education towards more deep and less surface approaches by means of implementing innovative teaching methods (e.g., Struyven et al. 2006; Wilson and Fowler 2005). We present a review study on students’ approaches to learning conducted within the context of one specific learning environment in which students’ approaches to learning have been studied extensively: problem-based learning (Loyens et al. 2013).

### Problem-based learning

Problem-based learning (PBL) is a student-centered instructional approach that is implemented at many universities worldwide. PBL students discuss professionally relevant problems in small groups. The problems are first discussed before any preparation or self-study has taken place to activate students’ prior knowledge. Because students’ prior knowledge is insufficient to fully understand the problem, questions (i.e., learning issues) are formulated for further individual self-study by the students in the group. After this individual self-study period (i.e., 2 or 3 days later), students gather again and discuss what they have learned and come to an answer to the formulated learning issues. The group discussion is facilitated by a teacher (i.e., so-called tutor) and is aimed to acquire knowledge, to better understand the problem, and to acquire skills to solve the problem (Barrows 1996). PBL does, on the one hand, actively engage students in their own learning and, on the other hand, includes many scaffolds to enhance student learning such as carefully designed problems and a group discussion facilitated by a tutor. The design of the PBL process (i.e., a pre-discussion of the problem to activate prior knowledge and formulate learning issues, an individual self-study time period, and a reporting phase in which different literature findings are discussed and integrated) is well aligned with current instructional design approaches that emphasize the importance of learning by means of whole problems in order to avoid fragmentation and encourage integration of knowledge, skills, and attitudes (Merrill 2012; Van Merrienboer and Kirschner 2013). Instead of learning small parts piece by piece, PBL emphasizes the integration of knowledge and skills. For example, discussing literature findings within a group makes that the answers to the learning issues become illuminated from different angles, since during the individual self-study period, students have—to a certain extent and within the boundaries of the problem’s topic—freedom to select and study their own literature resources. Besides learning content knowledge during the reporting phase, students also learn how to understand the underlying mechanisms of the problem at hand and hence, their problem-solving skills get trained at the same time. In other words, since students discuss relationships between concepts and principles, integrate different literature resources, apply these concepts and principles to the problems that are discussed in the group, and integrate knowledge and skills, PBL is assumed to encourage a deep approach to learning.

### The present study

The present review study is aimed at investigating the effects of PBL on deep and surface learning. We define deep learning in terms of students approaches to learning, reflecting both intentions or motives and actual strategies. We consider a deep learning approach as being intrinsically interested and aimed at trying to understand what is being studied. A surface approach is defined as an intention and strategy that is mainly aimed at rote learning and studying to pass the test. So, this review does start from a theoretical framework in which deep and surface learning approaches are seen as a combination of students’ intentions and accompanying learning activities, which are assumed to be related to the learning environment and are not seen as purely personal characteristics. In this review we focus on studies conducted in a problem-based learning environment. In line with earlier reviews on the effects of PBL (e.g., Dochy et al. 2003) we distinguish between either a curriculum wide or single course PBL implementation. In addition, when reviewing the relevant papers we will focus on the validity of the tools used to measure deep and surface approaches as well as the type of design or methodological quality of the studies. In this way, our study aims to meet the recommendations made by Dinsmore and Alexander (2012) mentioned earlier. The research questions addressed in this study are:

1. What are the effects of problem-based learning on students’ deep and surface approaches to learning?
2. Do the effects differ across (a) the context of the learning environment (single course vs. curriculum wide implementations of PBL), and (b) study quality (methodologically high level quality of studies vs. low/medium quality studies)?

## Methods

According to the Campbell Collaboration, a systematic review of the literature should include (1) clear criteria for inclusion, (2) a clear search strategy (3) systematic coding and (4) a systematic analyses of the included studies, using meta-analyses techniques were appropriate (www.campbellcollaboration.org). We will discuss below how we have taken these four recommendations into account in our review of the literature.

### Criteria for inclusion

Several criteria were defined for inclusion of studies in our review. First, the study should be conducted in a problem-based learning environment that is characterized by: (a) learning in small groups, (b) a teacher/tutor facilitating group learning, (c) the learning process is initiated by problems, and (d) new information is acquired through self-study (Barrows 1996). Second, each study should contain empirical data dealing with a deep or surface approach to learning. We did not restrict our studies to studies in which PBL curricula were compared with other curricula, nor did we restrict to quantitative studies; we included studies using different methodologies.

### Literature search

Studies published between 1900 and 2015 were searched. The following data-bases were used: EBSCO, PUBMED, and Web of Science. The keywords used were: problem based learning, PBL, problem oriented learning, POL, problem-based approach, problem-based learning program, and PBLP in combination with deep learning, deep-rooted learning, deep understanding, rote learning, surface learning, and superficial learning. The studies were selected based on title and abstract. Based upon reading these studies, 21 papers were included in this review. Reasons for rejection of papers were because of not reporting effects on deep or surface learning, not focusing on PBL as defined earlier, and not including data (book chapters/commentaries).

### Coding study characteristics

Based on our research questions, we developed a scheme to code and summarize all studies included. The following information was summarized: authors, journal, publication year, purpose, full PBL or hybrid curriculum, single course or curriculum wide implementation, study design, instruments, subjects, main conclusions, explanations of results, and suggestions for future research (see “Appendix”). The summaries were made by one of the authors (HM) and read by all other authors. The first author (DD) modified the summaries for those parts that were not yet clearly summarized in the opinion of the other authors. Subsequently, three authors (DD, SL, and DG) coded the papers based on the written summary. Each author coded whether the study dealt with PBL or a hybrid course/curriculum, whether the study dealt with a single course or curriculum wide implementation, whether it was a quantitative, qualitative or mixed-methods study, a one group (1 point) or experimental-control group design (2 points), a post-test only (1 point) or pre-post-test design (2 points), a longitudinal study (i.e., at least three measurement moments) (1) or not (0), whether the sample size was adequate (1) or not (0) (i.e., at least 40 subjects for the quantitative data), the instrument was tested to be reliable; e.g., reliability coefficients of .70 or higher (1), the instrument was tested to be valid; factor analysis confirmed the underlying factors (1). The overall study quality was rated based on the points that were received; a total score of 3 or lower was considered to be a study with low quality; a score of 4 and 5 as moderate, and a score of 6 till 8 as high quality. In addition, each study was scored in terms of its effect on deep learning: increase/positive (+), no effect (0) or decrease/negative (−). Similarly, each study was scored in terms of its effect on surface learning: increase/negative (+), no effect (0), decrease/positive (−). Finally, factors influencing approaches to learning were summarized in words. The coding was done by three authors (DD, SL, and DG). Disagreements were resolved through discussion.

### Synthesizing research

For our purposes, a systematic review of the literature was conducted accompanied by the vote counting method and the associated sign test (Cooper et al. 2009; Hedges and Olkin 1980). If the original studies reported the necessary information, also effect sizes were calculated for each individual study based on the standardized mean differences following Lipsey and Wilson (2001).

## Results

### What are the effects of PBL on deep and surface approaches to learning?

Table 1 provides an overview of the 21 studies. As can be seen in the final row of this Table 1, 17 studies were conducted in a full PBL environment, 3 in a hybrid PBL environment and 1 in a PBL computer environment. Furthermore, 12 studies were done in a curriculum wide PBL implementation and nine in a single course PBL environment. Table 1 also demonstrates that PBL does enhance a deep approach to learning in eleven studies. PBL does lead to a decrease in deep approach in four studies and has no effect on deep approach in another six studies. Furthermore, it is shown in Table 1 that PBL has no effect on a surface approach to learning in eleven studies. PBL does lead to a decrease in surface learning in six studies and an increase in four studies.

**Table 1:** Effects of PBL on deep and surface approaches to learning across full PBL or hybrid PBL and across studies conducted in a single course PBL environment versus a curriculum wide PBL implementation

Study nr	Full PBL or hybrid PBL	Single course or curriculum wide PBL	Deep aproach^a^ (ES)	Surface approach^b^ (ES)
1	PBL computer	Course	Increase (0.93)	Decrease (−0.50)
2	PBL	Curriculum	Decrease (−0.53)	Increase (0.50)
3	Hybrid	Curriculum	Increase (0.33)	No effect (−0.28)
4	PBL	Course	No effect (0.00)	No effect (0.13)
5	PBL	Course	Decrease (−0.38)	Increase (0.50)
6	PBL	Curriculum	Decrease (−0.36)	No effect (0.23)
7	PBL	Curriculum	Increase (0.16)	Increase (0.24)
8	PBL	Curriculum	Increase	Decrease
9	PBL	Curriculum	Increase (−0.41)	No effect (0.23)
10	Hybrid	Curriculum	No effect (0.44)	No effect (−0.45)
11	PBL	Course	No effect (0.00)	No effect (−0.03)
12	PBL	Course	Increase	No effect
13	PBL	Course	No effect	No effect
14	PBL	Course	Increase	Decrease
15	PBL	Curriculum	Increase (0.23)	No effect (0.10)
16	PBL	Curriculum	Decrease (−0.38)	Increase (0.50)
17	Hybrid	Course	No effect (−0.17)	No effect (−0.07)
18	PBL	Course	Increase (0.29)	No effect (−0.17)
19	PBL	Curriculum	Increase (0.21)	Decrease (0.36)
20	PBL	Curriculum	No effect	Decrease
21	PBL	Curriculum	Increase (0.50)	Decrease (0.00)
Total (*n* = 21)	PBL (*n* = 17) Hybrid (*n* = 3) Computer (*n* = 1)	Curriculum wide (*n* = 12) Single course (*n* = 9)	Increase (*n* = 11) Decrease (*n* = 4) No effect (*n* = 6)	Increase (*n* = 4) Decrease (*n* = 6) No effect (*n* = 11)

ES: effect size (Cohen’s d) calculated if possible based on Lipsey and Wilson (2001)

^a^Deep approach: increase/positive, no effect, decrease/negative effect

^b^Surface approach: increase/negative effect, no effect, decrease/positive effect

Table 2 presents the result of the vote-counts, sign test and effect sizes in order to give an answer on our first research question related to the main effects of PBL on deep and surface approaches to learning (Cooper et al. 2009). The vote count in Table 2 shows a positive tendency for the effects of PBL on deep learning with eleven studies of the 21 yielding a positive effect (i.e., a higher score or increase in the learning approach which we will label in the remaining of the text as ‘increase’) compared to four studies yielding a negative effect (i.e., a lower score or decrease in the learning approach which we will label in the remaining of the text as ‘decrease’). It should be mentioned, however, that this difference between studies fostering and lowering a deep approach to learning, was not statistically significant. The average effect size of .11 points towards a small positive effect of PBL on the deep approach.

**Table 2:** Main effects of PBL: vote counts and effect sizes

Outcome	Significance	Significance	No effect	Effect size
Deep approach^a^	11^ns^ increase	4 decrease	6	0.11
Surface approach^b^	6^ns^ decrease	4 increase	11	0.08

*Sign.* number of studies with an increase/decrease in deep and surface approach to study

*Studies (n)* the number of total non-independent outcomes measured

^ns^Two-sided sign-test is not significant at the 5 % level

As for the effects of PBL on surface learning approaches, eleven studies show no effect on surface learning, six studies show a lower score or decrease and four studies an increase in surface learning. Again, the two-sided sign-test was not significant for the number of studies decreasing and increasing a surface approach to learning and also the average effect size of .08 indicates that PBL has little effect on the surface approach.

### Does context or study quality impact deep learning?

In Table 3 the effects of studies reporting positive, negative or no effects on deep and surface approaches to learning across studies conducted in a curriculum wide PBL implementation (*n* = 12) and a single course PBL implementation (*n* = 9) are reported. The vote count indicated that for curriculum wide PBL implementations, seven studies showed an increase in deep learning, three studies led to a decrease in deep learning and two studies showed no effect. The effect size of .18 indicates a small effect of PBL in a curriculum wide implementation on students’ deep approach. Four studies indicated a decrease in surface learning, whereas three studies showed an increase in surface approach and another five studies no effect. However, the two-sided sign-tests were not significant for both the effects on deep and surface approaches to learning, meaning that the difference in number of studies reporting an increase and decrease in both deep and surface approaches to learning, is not statistically significant. Also the effect size of .08 gives an indication that a curriculum wide implementation of PBL has little effect on the surface approach.

**Table 3:** Effects of PBL curriculum wide and single course implementations: vote counts and effect sizes

Outcome	Significance	Significance	No effect	Effect size
Curriculum wide (*n* = 12)				
Deep approach	7^ns^ increase	3 decrease	2	0.18
Surface approach	4^ns^ decrease	3 increase	5	0.08
Single course PBL (*n* = 9)				
Deep approach	4^ns^ decrease	1 decrease	4	−0.05
Surface approach	2^ns^ decrease	1 increase	6	0.07

*Sign.* number of studies with an increase/decrease in deep and surface approach to study

*Studies (n)* the number of total non-independent outcomes measured

^ns^Two-sided sign-test is not significant at the 5 % level

The bottom part of Table 3 reports the effects on deep and surface approaches to learning across studies conducted in a single PBL course (*n* = 9). The vote count showed that four studies showed an increase in deep learning, one study led to a decrease on deep learning and four studies showed no effect. Table 3 also demonstrates that two studies showed a decrease in surface learning, one study gave evidence of an increase in surface approach and another six studies showed no effects. Similarly to the results for curriculum wide implementations, the two-sided sign-tests were not significant for both the effects on deep and surface approaches to learning. Hence, the difference in number of studies fostering or hindering both deep and surface approaches to learning was not statistically significant. However, for single course PBL implementations, the majority of the studies did not show an effect on surface approaches to learning. The effect sizes for both the deep (−0.05) and the surface (.07) approach are close to zero for the single course implementation of PBL.

In Table 4 the methodological quality of the studies is summarized. As can be seen from this table, the majority of the studies were quantitative studies (*n* = 18) and a minority were mixed-methods studies (*n* = 3). In terms of study designs, eleven studies involved experimental control group studies and ten studies used a one group design. In total, 14 pre-post test study designs were used and seven post-test only designs. Only one study was a longitudinal study with three measurement moments. The sample size was clearly above 40 in 16 out of 21 studies. The majority of the studies did make use of the Study Process Questionnaire developed by John Biggs and colleagues (11 studies). Only three studies reported about the validity of the instrument used and seven studies about the reliability of the data. In total, eight studies had a high overall study quality score (a score of 5, 6 or 7).

**Table 4:** Summary of methodological quality of the studies

	Design: quantitative qualitative mixed methods (MM)	Design: one group (1) or exp-control group (2)	Design: post only (1) or pre-post (2)	Sample size adequate no (0) yes (1) at least 40	Instru-ment	Valid no (0) yes (1)	Relia-ble no (0) yes (1)	Overall study quality^a^
1	Quantitative	One	Pre-post	Yes	LAQ	No	Yes	5
2	Quantitative	Exp-control	Pre-post	Yes	ASSIST	Yes	Yes	7
3	Quantitative	Exp-control	Post only	Yes	SIAL	No	No	4
4	Quantitative	One	Pre-post	Yes	SPQ	No	Yes	5
5	Quantitative	Exp-control	Post only	Yes	SPQ	No	No	4
6	Quantitative	One	Pre-post	Yes	SPQ	No	No	4
7	Quantitative	One	Pre-post	Yes	SPQ	No	Yes	5
8	Quantitative	Exp-control	Post only	Yes	SPQ	No	No	4
9	Mixed	Exp-control	Post only	Yes	ALSI	No	No	4
10^b^	Quantitative	One	Pre-post	No	ASSIST	No	Yes	5
11	Quantitative	Exp-control	Pre-post	Yes	SPQ	No	No	5
12	Quantitative	Exp-control	Pre-post	No	ALS	No	No	4
13	Quantitative	One	Pre-post	No	SPQ	No	No	3
14	Mixed	One	Post only	No	SPQ	No	No	2
15	Mixed	One	Pre-post	Yes	SPQ	Yes	Yes	6
16	Quantitative	Exp-control	Post only	Yes	SPQ	No	No	4
17	Quantitative	Exp-control	Pre-post	Yes	ASSIST	Yes	Yes	7
18	Quantitative	One	Pre-post	Yes	SIAL	No	No	4
19	Quantitative	Exp-control	Post only	Yes	LASI	No	No	4
20	Quantitative	Exp-control	Pre-post	Not given	ASSI	No	No	4
21	Quantitative	One	Pre-post	Yes	ASSI	No	No	4

^a^The overall study quality was calculated based on the scores received for the study design, sample size, validity and reliability of the data. There was one longitudinal study see ^b^ with three measurement moments, which received one extra score

The effects of PBL on deep and surface learning depending on study quality are mentioned in Table 5. For high-quality studies (*n* = 8), the vote count showed that three studies showed an increase in deep learning, one study led to a decrease on deep learning and four studies showed no effect. The effect size of .13 points towards a small positive effect. With respect to surface learning, two studies showed a decrease, one study an increase and five no effect. The effect size was with −.01 close to zero. Similar to the results regarding the scale of PBL implementation, the two-sided sign-tests were not significant for both the effects on deep and surface approaches to learning.

**Table 5:** Effects of PBL depending on study quality: vote counts and effect sizes

Outcome	Significance	Significance	No effect	ES
High-quality studies (*n* = 8)				
Deep approach^a^	3^ns^ increase	1 increase	4	0.13
Surface approach^b^	2^ns^ decrease	1 decrease	5	−0.01
Medium–low quality studies (*n* = 13)				
Deep approach^a^	7^ns^ increase	4 decrease	2	0.07
Surface approach^b^	6^ns^ decrease	1 increase	6	0.17

*Sign.* number of studies with a significant increase or decrease in deep and surface approach to study

*Studies (n)* the number of total non-independent outcomes measured

^ns^Two-sided sign-test is not significant at the 5 % level

In the bottom part of Table 5, results are mentioned for low and medium quality studies (*n* = 13). Seven studies gave evidence of an increase in deep learning, four studies led to a decrease on deep learning and two studies showed no effect. The effect size of .07 indicates there is no or a very small effect. For surface approaches to learning, six studies showed a decrease, one study showed an increase and six studies showed no effects. For medium–low quality studies, although almost half of the studies found no effect on surface approaches to learning, the effect size of .17 points towards a meaningful effect.

Again, the two-sided sign-tests were not significant for both the effects on deep and surface approaches to learning, meaning no differences could be found in the number of studies showing an increase versus a decrease for both deep and surface approaches to learning.

## Conclusion and discussion

This review was aimed at investigating the effects of PBL on deep and surface approaches to learning. The studies included were all conducted within the specific context of PBL and most of the studies used Biggs’ theoretical framework to measure deep and surface processing. Dinsmore and Alexander (2012) made a plea to study deep learning approaches from a clear theoretical framework and within a specific context; a specific learning environment. We addressed these points in this review. The review demonstrated that eleven of the 21 the studies give indications that PBL does encourage a deep approach to learning and in eleven of the 21 studies measuring surface learning, PBL had no effect on a surface approach. As also indicated by the effect sizes, PBL does seem to enhance deep learning to some extent (ES = .11) and has less effect on surface learning (ES = .08). Furthermore, this review demonstrated that differences in effects between the studies could be partly explained by differential characteristics of the environment in which the PBL studies were conducted (a curriculum wide implementation has a more positive impact on students’ deep approach (ES = .18) compared to a single course (ES = −.05) implementation), but not by study quality.

The mechanisms through which PBL is assumed to enhance deep learning are active and self-directed learning. PBL is considered an active form of learning, since students need to analyze, compare, contrast, and explain information (Serife 2011). They are actively involved in their learning process because they themselves need to develop and explain hypotheses for the problem at hand and search for evidence for these explanations and hypotheses, using various literature and other learning resources (Gurpinar et al. 2013). Self-directed learning comes into play in PBL since students take responsibility over their own learning. They have, to a certain degree and within the boundaries of the problem, the freedom to select their own resources to answer the learning issues, which gives them ownership over their learning. Eleven out of the 21 studies included in this review demonstrate that PBL does foster deep learning (ES = .11). This effect is possibly mediated through intrinsic motivation. A recent PBL study in which having the freedom to choose literature resources (i.e., self-directed condition) from a set was compared to a condition in which two literature resources were given to students, indeed demonstrated that students in the self-directed condition scored higher on autonomous motivation (Wijnia et al. 2015), giving evidence for the relationship between self-directed learning and autonomous/intrinsic motivation.

The findings of this review also indicate that PBL has little effect on surface learning in eleven out of 18 studies (ES = .08) measuring surface learning. Is this good news or not? It could be argued that this finding is in a way a positive effect too. Nevertheless we should also take into account that in some situations a surface approach or perhaps better a combination of a deep and surface approach should best be used to learn effectively (Dinsmore and Alexander 2012). A high perceived workload will more likely result in surface approaches to studying and might be detrimental for deep learning. Students who perceive the workload as high in their learning environment are more likely to display a lack of interest in their studies as well as exhaustion. This is particularly true for beginning PBL students (Litmanen et al. 2014). Another factor that can lead to more surface learning is the assessment methods used. If the assessment is perceived as not rewarding deep learning, students will rely on surface learning. Therefore, the role of assessment is important to take into account in studies on SAL. Entwistle et al. (2003, p. 90) state in this respect that research findings vary “due to differences in the extent to which understanding is explicitly rewarded in the assessment procedure”. A qualitative study by Al Kadri et al. (2009) under PBL medical students confirmed indeed that students adapt their approaches to studying to the assessment demands (i.e. type of assessment and weight accorded to it). Scouller (1998) and Jensen et al. (2014) demonstrated that students were more likely to employ a deep approach when studying for assignment essays, which they perceived as measuring higher levels of cognitive processing, compared to a multiple choice assessment.

Although most studies demonstrate that PBL does enhance deep learning and has no effect on surface learning, this review also shows that studies often result in ambiguous and inconsistent findings as is also concluded by Dinsmore and Alexander (2012). One reason is that only three studies out of 21 studies reported about the validity of the data and eight about the reliability of the data. Often evidence of validity was lacking as concluded before by Dinsmore and Alexander (2012). Within this review we investigated deep learning within a specific context, being PBL. Although the studies demonstrated a trend towards a positive effect on deep learning and no effect on surface learning, findings differed across studies which could indicate that PBL is applied differently across the different studies, even although we included only studies in this review that met our definition of PBL. In addition, in one study it was argued that students already displayed high scores on deep learning due to which it might be difficult to further improve deep learning (Reid et al. 2005).

This review has several limitations. First of all, the studies included in this review only made use of self-report data; actual student behaviors were not measured and could differ from students’ self-perceptions. Next, the relationship with academic achievement was not considered in this review. Further, the number of longitudinal studies and qualitative studies was limited and some studies included only one group (i.e., no control group) or only post-test data (i.e., no pre-test data) due to which no clear comparisons could be made. As mentioned, not all studies included reported data about the validity and reliability of the instruments used to measure deep and surface processing, although the majority of the studies used previously validated instruments. Not all the studies included in the review reported the necessary information to calculate effect sizes. Hence, effect sizes of only 16 studies were included and aggregated across different study designs. For future research, more longitudinal studies are needed to determine the long terms effects of PBL on deep and surface learning, as well as experimental studies with a control group and pre- and post measurements that can give better insight in the actual changes in students’ deep and surface processing. Longitudinal studies provide opportunities to measure how approaches to learning might differ over time, although it should be taken into account that characteristics of the learning environment may also vary over time. Qualitative studies are needed as well since they could give us better insight in why and how PBL does or does not enhance deep and surface processing. Finally, future studies should report validity and reliability data of the instruments used to measure deep and surface processing.

## Appendix with summary of the 21 studies

**Table:**

	References	Journal	Year	Objectives	Design	Instrument	Main conclusion
1*	Serife (2011)	Current issues in education	2011	To investigate the effects of computer supported PBL on students’ approaches to learning	One group pre- and post-test design. PBL implementation during 5 weeks	LAQ	Problem based learning has a significant effect on adopting a predominantly deep approach to learning by students and a negative effect on adopting surface approach to learning
2*	Papinczak et al. (2008)	Advances in Health Sciences Education	2008	To determine the influence of metacognitive activities—self and peer-assessment—within the PBL tutorial environment on the development of deep learning approach, reduction in surface approach, and enhancement of individual learning self-efficacy	Control group pre-test, post-test design was implemented	ASSIST	Over the course of first-year medical studies, students lose self-efficacy and move away from deep-strategic learning approaches towards more surface approaches. The program of metacognitive activities failed to reverse this trend. The substantial swing towards surface learning raises questions about the perceived capacity of PBL curricula to promote deep approaches to learning in dense curricula
3*	Abraham et al. (2008)	Advances in Physiology Education	2008	To study the differences in learning approaches to physiology of undergraduate medical students in a partially PBL and non-PBL oriented curriculum	Control group post-test only design. PBL curriculum from September 2006 admissions onward	SIAL	Scores for deep and strategic approaches of PBL students were found to be significantly higher compared with NPBL students. No difference between PBL and NPBL in surface approach
4*	Wong and Lam (2007)	Research on social work practice	2007	To evaluate the effects of problem-based learning (PBL) in social work education	One group pre-test post-test design. 132s-year social work students who were spread across the 3 academic years of 2000–2001, 2001–2002, and 2002–2003	SPQ + R-SPQ	The results indicated positive learning outcomes, with the most significant gains occurring in knowledge and lesser gains being made in skills and values. No significant positive gain in deep learning and no significant change in surface learning. Surface approach is negatively correlated with learning outcomes. The findings suggest that students with deep learning motives and approaches reap the most benefit from PBL. The switch to the PBL mode increased the students’ workload and did not necessarily result in a deeper learning approach for all of them
5*	Segers et al. (2006)	Studies in Educational Evaluation	2006	To determine if students in a redesigned course, firstly, hold different perceptions of the assessment demands and, secondly, adjusted their learning strategies towards deeper learning	Control group post-test only design—two subsequent cohorts of second-year students	SPQ	Contrary to expectations, the students in the original assignment-based (ABL) course adopted sign. more deep-learning strategies and sign. less surface-learning strategies than the students in the problem-based (PBL) course. Additionally, the results show clearly that the students who express their intentions to employ a certain learning strategy perceive the assessment demands as such and actually employ a related learning strategy
6*	Groves (2005)	Advances in Health Sciences Education	2005	To assess the influence of graduate-entry PBL curriculum on individual learning style and investigate the relationship between learning style, academic achievement and clinical reasoning skill	One group pre-test post-test design	SPQ	Net shift towards a more surface approach over the period of the study (but not significant). Significant decrease in deep-learning scores
7*	Mok et al. (2009)	International journal of speech-language pathology	2009	To better understand the relationship between student learning approaches and academic performance in a problem-based learning (PBL) curriculum	One group pre-test post-test design Cross sectional research design (comparison of 3 cohorts)	R-SPQ-2F	Exposure to PBL led to significant increase in DA (deep approach) and SA (surface approach) to learning during an academic year for students in years 1–3. Students who did well in a PBL examination showed a much stronger DA than SA to learning, while students who performed less well showed a smaller difference between DA and SA to learning
8*	Gurpinar et al. (2013)	Advances in Physiology Education	2013	Determine the satisfaction of medical students with problem-based learning (PBL) and their approaches to learning	Control group post-test only design (three curricula were compared; one full PBL vs. two hybrid) Cross-sectional	R-SPQ-2F	Of the study group, 64.6 % were found to adopt a deep approach to learning, and it is confirmed that these students were reasonably more satisfied with PBL. When the three different curricula were compared in terms of student satisfaction with PBL among surface and deep learners, no significant differences among surface learners in different curricula was found in terms of satisfaction. However, in the full PBL curriculum a higher percentage of students adopted a deep approach and a lower percentage a surface approach as compared to the other curricula (control groups)
9*	Grant et al. (2012)	BMC Research Notes (Biomedicalcentral)	*2012*	To compare the effect of context on learning at different UK medical schools, schools with conventional and PBL curricula	Control group post-test only design Mixed method—two stages, first qualitative phase and then, quantitative phase where findings from the qualitative phase were tested	ALSI (Entwistle)	Students with PBL curriculum scored significantly higher for reflection in learning, self-efficacy in self-directed leaning and for deep approach to learning. Students surface approach did not differ significantly
10*	Reid et al. (2012)	Medical Education Online	2012	To investigate the hypothesis that the redesigned curriculum was successfully promoting a deep approach to learning and studying and deterring a surface approach in undergraduates during years 1–5 of a medical degree program	One group pre-test post-test design Quantitative Longitudinal	ASSIST	Medical students have high scores for deep and strategic approaches to learning and studying and lower scores for a surface approach, but that, even when efforts were made to promote deep approach, little significant change in these scores occurred during the whole of the medical degree program, apart from some tendency for the surface approach to lessen. Either their approaches are not susceptible to change or else the learning environment may need to alter more drastically than hitherto
11*	McParland et al. (2004)	Medical Education	2004	To measure the effectiveness of a problem-based learning course compared to traditional teaching in undergraduate psychiatry	Control group pre-post test design. A PBL psychiatry course versus a lecture-based psychiatry course	SPQ	The PBL attachment/course resulted in significantly better examination performance than did the traditional teaching course. No differences in surface, deep or strategic learning before and after the course were found. No differences between the two courses. Students were significantly more successful in the examinations if they had received the PBL course, were female, and used deep and strategic learning
12*	Selçuk (2010)	International Journal of the Physical Sciences	2010	To evaluate the effects of (PBL) method on students’ achievement in and approaches and attitudes towards an introductory physics course	Control group pre-test post-test design. One control group (or traditional lecture-based instruction group) and one experimental group (PBL group)	ALS	The results indicated that the problem-based learning method encouraged the students’ deep approach to learning as compared to the control group (sign), and also improved interest (a component of attitude) towards the physics course. The results also signalled that PBL-based physics instruction impacted the students’ achievement in physics positively. No sign. difference between PBL and control group in terms of surface approach were found before and after the course
13*	Kieser et al. (2005)	European Journal of Dental Education	2005	To analyze the influence of context on students’ approaches to learning	One group pre-test post-test design. Low N!	R-SPQ-2F	Those who entered the course with a surface approach (*n* = 5) left with a deep-learning approach, and quality learning outcomes. There were 7 students who started the course with a deep-learning approach and cohesive conception and had a deep at the end (no change). There were twowho moved from deep to surface. Test results were better for students with a deep approach and worse for students with a surface approach
14*	Schultz and Christensen (2004)	European Journal of Engineering Education	2004	To evaluate the implementation of the highly structured seven-step problem-based learning (PBL) procedure as part of the learning process in a human–computer interaction (HCI) design course	One group post-test only design. Low N! Mixed methods (both qualitative and quantitative methods)	SPQ-modified	The qualitative and quantitative evaluation showed that students took responsibility for their own learning. The quantitative evaluation shows that PBL clearly stimulated the students to take a deep approach to learning and not a surface approach (i.e. the mean scores on items related to deep approach differed from the items dealing with a surface approach; in favour of the deep approach [1.4 difference, scale 1–5)]
15*	Tiwari et al. (2006)	Nurse Education Today	2006	To evaluate the effect of PBL on students’ approaches to learning in clinical nursing education	One group pre-test post-test design	R-SPQ-2F	Study provides empirical support for the suggestion that PBL promotes a deep approach to learning. The R-SPQ-2F scores indicated that for the deep approach to learning, the post-test mean score was significantly higher than at the pre-test. No significance was observed between the pre-test and post-test mean scores for the surface approach to learning
16*	Nijhuis et al. (2005)	Learning Environ-ments Research	2005	To determine if students, firstly, perceived the redesigned course as being more challenging and, secondly, adjusted their learning strategies towards deeper learning	Control group post-test only design. Quantitative methods comparing two groups	SPQ-adapted	The results indicated that the students from the redesigned course showed a higher degree of surface learning and a lower level of deep learning than the students from the assignment-based learning course
17*	Reid et al. (2005)	Medical Teacher	2005	To determine to what extent the early medical course succeeded in promoting a deep approach and deterring a surface approach to learning	Control group pre-test post-test design. Longitudinal study	ASSIST	The results are remarkably consistent from cohort to cohort with relatively high scores for deep (60 out of 80) and strategic approaches and lower for surface (45 out of 80). Disappointingly, the students’ learning approaches did not show any increase in deep approach during year 2, also no change in surface approach was reported
18*	Adiga and Adiga (2010)	Biomedical Research	2010	To study the changing pattern of learning approaches to pharmacology adopting PBL by undergraduate students of an Indian medical school	One group pre-test post-test design. Quantitative method. Mean scores of surface, deep and strategic approaches of students during pre-PBL (end of 2^nd^ block) and post-PBL phase (end of the 4^th^ block) were compared	SIAL	Scores for deep approaches of students in post-PBL phase (3^rd^ and 4^th^ blocks) were found to be significantly higher compared with pre-PBL phase. The score for the surface and strategic approaches did not differ significantly between the two phases even though there was a small change
19*	Newble and Clarke (1986)	Medical Education	1986	To explore the relationship between educational context and approach to learning	Control group (i.e., traditional medical school vs. a PBL medical school) Posttest only	Lancaster Approaches to Studying Inventory	PBL students appear to have an approach to learning which more closely approximates the aims of most medical schools (i.e., high on deep approach and low on surface)
20*	Coles (1985)	Medical Education	1985	To compare PBL and non-PBL students in their approaches to learning	Control group Pre-Posttest design (on entry and after one year)	Short Inventory of Approaches to Studying	The approaches to studying of students at the conventional school appcar to be detrimentally influenced by the experience of the first year Those of PBL students do not, and probably are improved
21*	De Volder and De Grave (1989)	Medical Education	1989	To investigate how the introductory phase of a PBL medical program affects the study methods of students	Pre-posttest design (on the first day of academic year and again after the introductory period, i.e., 6 weeks)	Short inventory of study Approaches (Entwistle 1981)	Results indicate that approaches to learning are made desirable by the training in PBL, but are not desirable on entry
